# The Alberta Quality Assessment Tool: Risk of Bias (AQAT:RoB) for the Evaluation of Medical Large Language Model Question-Answer Studies: Development and Pilot Validation

**DOI:** 10.2196/87057

**Published:** 2026-04-08

**Authors:** Carrie Ye, Joseph Ross Mitchell, Daniel C Baumgart, Zechen Ma, Angela Lim Fung, Daniela Garcia Orellana, Juel Chowdhury, Abdullah Abass, Steven Katz, Jacob L Jaremko, Pierre Boulanger, Claire E H Barber, Gillian Lemermeyer, Hosna Jabbari, Lili Mou, Maryam Mirzaei, Mary Waithera Beckett Githumbi, Puneeta Tandon, Randy Goebel, Rhys Clark, Whitney Hung, Marjan Abbasi, Farhad Maleki, Scott Klarenbach, Mohamed Abdalla

**Affiliations:** 1 University of Alberta, 8-130 Clinical Sciences Building, 11350 83 Ave NW, Edmonton, AB, T6G2G3, Canada, 1 7804927002, 1 7804926088; 2 Arthritis Research Canada, Vancouver, BC, Canada; 3 Alberta Health Services, Edmonton, AB, Canada; 4 Alberta Machine Intelligence Institute, Edmonton, AB, Canada; 5 University of Toronto, Toronto, ON, Canada; 6 University of Oxford, Oxford, United Kingdom; 7 Queen's University, Kingston, ON, Canada; 8 University of Calgary, Calgary, AB, Canada; 9 NAIT Applied Research, Edmonton, AB, Canada

**Keywords:** risk of bias, quality assessment, large language model, question-answer studies, Alberta Risk of Bias Assessment Tool for LLM-QA studies, AQAT: RoB, chatbot, artificial intelligence

## Abstract

**Background:**

Despite the transformative potential of large language models (LLMs) in health care, the rapid development of these tools has outpaced their rigorous evaluation. While artificial intelligence–specific reporting guidelines have been developed to address standardized reporting of artificial intelligence studies, there is currently no specific tool available for risk of bias assessment of LLM question-answer (QA) studies. Existing risk-of-bias tools for medical research are not well suited to the unique challenges of evaluating LLM-QA studies, which creates a critical gap in assessing their safety and effectiveness.

**Objective:**

This study aims to develop the Alberta Quality Assessment Tool: Risk of Bias (AQAT:RoB) for LLM-QA studies to systematically evaluate the validity and risk of bias in LLM-QA studies.

**Methods:**

We conducted 2 literature reviews. The first was on quality assessment tools for LLM-QA studies, and the second was on LLM-QA studies, which informed the first draft of the AQAT:RoB. The draft AQAT:ROB was further refined through a prespecified iterative process of modified Delphi, consensus meeting, and validation. The first Delphi process occurred between May 1 and May 20, 2025, and the first consensus meeting was held on May 22. The first round of validation was completed by 4 evaluators, who were not part of the consensus meeting, on 16 randomly selected studies. As this first round of validation surpassed our a priori threshold of ≥80% agreement and a Cohen κ of ≥0.61 between evaluators, no further rounds of development and validation were undertaken. A second Delphi process occurred between February 20 and February 23, 2026, to vote on postpilot changes in response to peer review.

**Results:**

The AQAT:RoB consists of 5 high-level domains (Questions, Reference Answers, LLM Answers, Evaluators, Outcomes). These domains are subdivided into 9 subdomains. Each subdomain includes at least one “Support for Judgment” and at least one “Type of Bias” and is to be rated “low,” “high,” or “unclear” for risk of bias. A pilot evaluation was completed by internal validators who were not part of the consensus discussion and were asked to complete the AQAT:RoB form for each assigned study. Each of the 16 studies was evaluated by 2 evaluators independently. Pilot validation showed a percent agreement of 86.1% and a Cohen κ of 0.70 between assessors.

**Conclusions:**

The AQAT:RoB demonstrates promising initial reliability for assessing the validity or risk of bias in LLM-QA studies. The tool will benefit from future refinements, external validation, and periodic updates to keep pace with evolving technology.

## Introduction

Large language models (LLMs) represent a significant technological advancement with transformative potential across various sectors, including health care. Their capabilities in processing and generating human-like text have led to their rapid emergence as tools capable of assisting in complex medical activities, such as disease diagnosis, clinical decision-making, and even administrative tasks, such as writing prescriptions or assigning billing codes [1]. As these sophisticated tools become more integrated into health care ecosystems, robust and rigorous evaluation of their efficacy, safety, and utility is paramount. A critical component of this evaluation involves human assessments, where the performance, usability, and impact of LLM question-answer systems (LLM-QA) such as medical chatbots are gaged through interactions with health care professionals, patients, or simulated users [2,3].

However, the quality of these human evaluation studies varies significantly, with a systematic review indicating that only 5% of studies used real patient care data for LLM evaluation, which can significantly impact the trustworthiness and generalizability of their findings [2]. Without a systematic approach to evaluating the quality and risk of bias in studies assessing LLM-QA, the rapid pace of development could outpace the generation of reliable evidence regarding their actual utility and safety in real-world clinical scenarios, potentially leading to premature or even harmful adoption.

Existing tools for assessing risk of bias, such as the Cochrane Risk of Bias 2 tool (RoB 2) [4] for randomized studies, the Quality Assessment of Diagnostic Accuracy Studies 2 (QUADAS-2) [5] for diagnostic test studies, the Newcastle-Ottawa Scale (NOS) [6], and the Risk of Bias in Non-randomized Studies - of Exposures tool (ROBINS-E) [7] for nonrandomized studies, are foundational in their respective areas but fall short when applied to the unique methodological and reporting challenges inherent in human evaluation studies of LLMs. While artificial intelligence (AI)–specific quality assessment tools exist, such as the Prediction model Risk of Bias Assessment Tool + AI (PROBAST-AI) [8] and APPRAISE-AI [9], these focus on studies of prediction models using machine learning and are not applicable to LLM-QA studies.

The burgeoning interest in the field is evident from the notable surge in studies pertaining to LLM medical chatbots published in recent years, underscoring the topic’s emerging relevance and the urgent need for robust evaluation methodologies [10,11]. AI-specific reporting guidelines have been developed to address standardized reporting of AI studies [9,12-15], including a reporting checklist specifically for chatbot health advice studies, CHART (Chatbot Assessment Reporting Tool) [13]. However, transparent and comprehensive reporting is only one aspect of quality assessment—the other being assessment of risk of bias.

There is currently no tool available to assess the risk of bias in LLM-QA studies. This gap creates a significant challenge for researchers, clinicians, and policymakers attempting to synthesize evidence and make informed decisions about the integration of medical LLM-QA systems. Without a comprehensive and tailored risk of bias assessment tool, the risk of misinterpreting findings, perpetuating methodological flaws, and drawing unsubstantiated conclusions from human evaluation studies is high. To address this knowledge gap, we took a pragmatic but systematic approach to develop and validate the Alberta Quality Assessment Tool: Risk of Bias (AQAT:RoB) for LLM-QA studies for the systematic and comprehensive assessment of the risk of bias in medical LLM-QA studies, addressing aspects unique to this emerging field. The tool is intended to evaluate the quality of studies that involve human participants in assessing the outputs of AI models that utilize natural language interactions.

## Methods

### Overview

**Figure 1.**
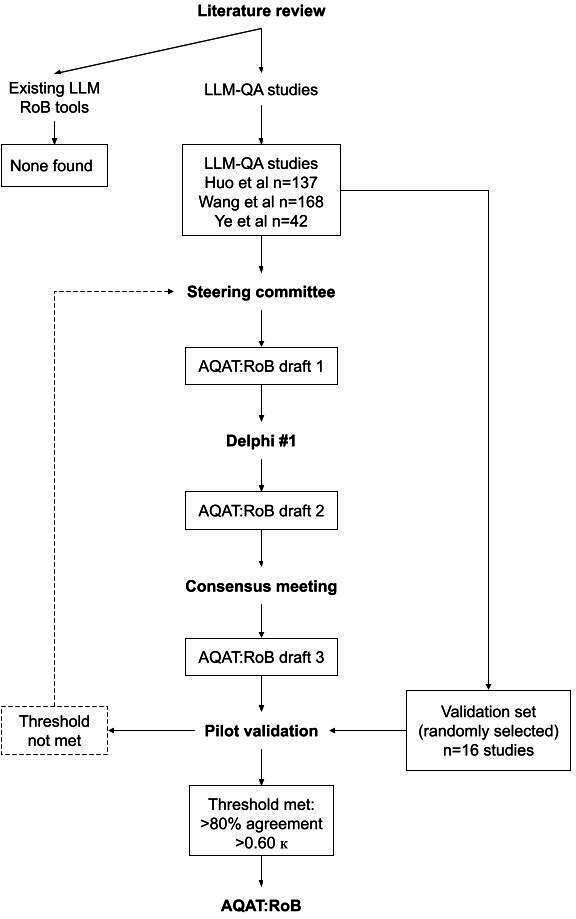
Alberta Quality Assessment Tool: Risk of Bias (AQAT:RoB) development and validation. This figure outlines the development and pilot validation process (April-September 2025) that was a priori determined and followed to create the AQAT:RoB. The dotted line implies a possible path that would have been followed had the agreement threshold not been met (though this was ultimately not required). LLMs: large language models; LLM-QA: LLM question-answer; RoB: risk of bias. LLM-QA studies include [10,11] and a forthcoming systematic literature review of studies evaluating LLMs for patient-facing health information (protocol registered with PROSPERO; CRD42023461630 [16]).

The AQAT:RoB development and validation started with the 2 literature reviews. The first was on quality assessment tools for LLM-QA studies, and the second was on LLM-QA studies, which informed the first draft of AQAT:RoB, which went through an iterative process of modified Delphi, consensus meeting, and validation, until our a priori threshold for interrater agreement was met (Figure 1).

### Ethical Considerations

The University of Alberta’s Research Ethics Board deemed that this project meets one of the conditions described under Chapter 2 of *Tri-Council Policy Statement: Ethical Conduct for Research Involving Humans* (2022) [17] as an activity that does not require Research Ethics Board review. The AQAT:RoB has been registered with the LATITUDES Network, which was established to increase the robustness of evidence synthesis by improving the process of validity (risk of bias) assessment [18].

### Literature Review

We conducted a search query in PubMed in April 2025 to look for existing risk of bias assessment tools specific to LLM-QA studies. The query searched for all studies that contained either of the terms “risk of bias” or “quality assessment” with any of the terms “large language models,” “generative AI,” or “chatbot” [(“risk of bias” OR “quality assessment”) AND (“large language models” OR “generative AI” OR “chatbot”)]. We limited the search to English studies published in the last 5 years and retrieved 91 results. The search was updated in June 2025, which retrieved 113 studies, and again in September 2025, which retrieved 149 studies. None of these searches included tools for assessing validity or risk of bias in LLM studies. Most studies pertained to the use of LLMs to perform risk of bias assessments. Around the time of each PubMed search, the LATITUDES Network library [19] of risk of bias assessment tools and tools in development was searched for tools that pertained to LLM studies; none were found.

We conducted a literature review of LLM-QA studies to (1) inform the development of AQAT:RoB and (2) find studies for validating the AQAT:RoB. Our group had already conducted, with the assistance of an experienced librarian (DCB), a systematic literature review of studies evaluating LLMs for patient-facing health information (manuscript in progress, protocol registered with PROSPERO [CRD42023461630]) [16]. Medline, Embase, Web of Science, CINAHL, PsycINFO, and Google Scholar were searched for studies published up to July 5, 2023, and then updated to March 7, 2024, limiting searches to the last 10 years prior to the search (Multimedia Appendix 1). Searches were limited to the English language but not to geographic regions. All citations were imported into Covidence for duplicate removal and screening. Abstracts and full texts were each screened independently by 3 reviewers (ZM, ALF, DGO). Disagreements were resolved through a third reviewer (CY). A total of 8943 records were identified, including 2798 duplicates (Multimedia Appendix 2). In total, 6145 titles and abstracts were screened with 327 found to be relevant for full-text review. Of these 327 full texts, 40 were deemed to be original research studies pertaining to LLMs for patient education (Multimedia Appendix 3).

We found 2 very recently published systematic reviews by Wang et al [11] and Huo et al [10], which, along with our systematic review [16], we felt covered the breadth of LLM-QA studies and were thus sufficient to inform the development and validation of the AQAT:RoB. In the systematic literature review conducted by Huo et al [10], their search of MEDLINE, Embase, and Web of Science from inception to October 27, 2023, resulted in 137 eligible studies evaluating the performance of generative AI-driven chatbots. They found that key aspects of internal validity, such as standardized evaluation process, blinding of evaluators, or reference standards, were not well described or simply not included in the studies [10]. Wang et al [11] queried PubMed, Embase, Web of Science, and Scopus from inception until October 14, 2024, and found 168 studies on the accuracy of LLMs when answering clinical questions (although one of these studies has since been retracted). They performed a risk of bias assessment using the NOS [6,11]. Acknowledging the limitations of using the NOS tool given the limited relevance to these studies, they found that only 40 (23.8%) of 168 studies were assessed as having a low overall risk of bias [11].

### Candidate Item List Generation

The initial list of items for the AQAT:RoB assessment tool was drafted by CY and MA by reviewing the results and studies of the 3 recent systematic literature reviews [10,11,16] to identify potential sources of bias in LLM-QA and by adapting existing foundational risk of bias assessment tools, including RoB 2 [20], NOS [6], QUADAS-2 [21], and ROBINS-E [7]. The steering committee (CY, MA, JRM, DCB) further developed the initial list of items to form the first draft of the AQAT:RoB (7 domains, 12 sources of bias, 15 supports). This first draft was used in the modified Delphi procedure described below.

### Recruitment of Delphi Panelists

To identify participants for the modified Delphi process, the steering committee recruited medical AI experts through the Alberta Machine Intelligence Institute [18,22], the University of Alberta AI + Health Hub [23], and the University of Calgary’s Centre for Health Informatics [24]. Interested participants were asked to complete an intake questionnaire regarding demographics, training, and related expertise. All applicants were screened, and panelists were selected by the steering committee to ensure appropriate expertise and diversity and representation on the Delphi panel. In total, there were 19 Delphi panelists, including clinicians (8), computer scientists (8), methodologists (4), researchers (qualitative and quantitative, 17), journal editors (2), and patient partners (2). All Delphi participants were instructed to watch 2 videos that provided background information on risk assessment tools prior to initiating the Delphi process.

### Modified Delphi Process

The modified Delphi process occurred between May 1 and May 20, 2025. The steering committee created the Delphi survey using Google Forms. Each participant responded to the survey individually. Participants were asked to rate each item’s potential as a source of bias on a 5-point scale (from 1=“Not a potential source of bias” to 5=“High potential source of bias”). If participants selected either 1=“Not a potential source” or 2=“Unlikely to be a potential source,” they were prompted to provide a short explanation. Participants were also encouraged to use the free-text boxes that followed each item to identify any missing potential sources of biases, questions to identify biases (ie, supports), or types of bias identified by listed supports. The threshold for removing items was more than 50% (10/19) of the participants voted the item as 1=“Not a potential source” or 2=“Unlikely to be a potential source,” and the threshold for retaining items was less than 50%. Participants were not able to see other participants’ votes or comments.

### Changes From the Delphi Process

Most items (8 of 12) were rated highly for inclusion (ie, more than 70% (14/19) rated as a potential source of bias). Items rated poorly for agreement or for potential source of bias, and all comments provided by the participants in the free-text boxes were considered for changes (eg, removal, modification, or merging). Based on feedback from participants, no domains or items were added or removed, but modifications were made to 5 items.

### Consensus Meeting

An online consensus meeting was held on May 22, 2025, chaired by the steering committee. All panelists were invited except for 2 (S Katz and JLJ) who were excluded from the discussion to serve as adjudicators in the validation phase. The consensus meeting was chaired by the steering committee, and 16 participants attended the synchronous consensus meeting. During this meeting, participants were presented with the initial version of texts; suggested modified versions of the text incorporating the feedback from the modified Delphi process, as well as summary statistics of ratings; and provided comments. During the meeting, each potential source of bias was discussed until consensus was reached on inclusion, type of bias(es), and wording. Once consensus was felt to be reached based on the panel discussion, a formal vote was taken, and unanimity was required before moving on to the next item. At the end of each domain, the panel was asked to discuss if there were any additional potential sources of bias pertaining to that domain.

### Changes From the Consensus Meeting

The discussions during the consensus process resulted in multiple changes. There was robust discussion about how granular the “Types of biases” should be, with the group arriving at the conclusion that for the sake of utility and widespread applicability, we would aim for a high-level description of the types of biases. This change affected 5 of 12 “Potential Sources of Bias.” Furthermore, there was an addition of “Support for Judgment” for the domain “Performance Metrics.”

### Pilot Validation

We piloted the AQAT:RoB on 16 studies [25-40], randomly selected from the 319 studies (after removal of duplicates) identified in 3 recent systematic literature reviews [10,11,16] (Multimedia Appendix 4). Random selection was facilitated by listing all 319 studies in alphabetical order of the first author’s last name and then using a random number generator (between 1 and 319) to select the 16 studies [41]. Four evaluators (S Katz, JLJ, JC, AA) were asked to complete the AQAT:RoB form for each assigned study. None of the 4 evaluators were part of the consensus discussion, but S Katz and JLJ were on the modified Delphi panel. JC and AA were not part of the tool development process prior to the validation step. All the evaluators were physicians across various specialties (rheumatology, radiology, public health, and primary care). Each of the 16 studies was evaluated by 2 evaluators independently. Evaluators were not provided with any standardized training in order to obtain the most conservative estimates of agreement.

We set an a priori threshold of >80% agreement and a Cohen κ of ≥0.60, which would demonstrate substantial agreement per Landis and Koch’s [42] classification system. If we reached this threshold, no further rounds of changes or consensus would be undertaken. If we did not reach this threshold, we planned to pursue further rounds of modified Delphi or consensus or validation until this threshold was achieved (Figure 1).

### Protocol Deviations in Response to Peer Review

The steering committee proposed moving the Reporting and Conflict of Interest domains to “Additional consideration” for the Delphi panel after the first round of validation was completed. The rationale was that Reporting was better assessed by stand-alone reporting checklists, and while reporting may lead to an unclear risk of bias judgment, it does not represent a mechanistic bias similar to the other domains in the AQAT:RoB. Similarly, Conflict of Interest may be a predictor or source of bias rather than a distinct mechanism of bias. We set the voting threshold to make this change at 70% for the Delphi panel and planned to conduct another round of validation if the overall percent agreement and Cohen κ after removing these 2 domains did not reach our a priori threshold of >80% agreement and Cohen κ of >0.60. Note that 100% of the panel voted in agreement with this change.

## Results

### Validation Results

After the first modified Delphi and consensus panel, we met our threshold with a percent agreement of 82.8% and a Cohen κ of 0.63 (calculated across all items; Multimedia Appendix 5). After the second modified Delphi, in response to peer review, during which 2 domains were removed, the percent agreement was 86.1% and Cohen κ was 0.70. The domain with the highest level of agreement was Question Selection (agreement: 93.8%, κ: 0.86), while the domain with the lowest level of agreement was LLM Answer Selection (agreement: 68.8%, κ: 0.30).

### AQAT:RoB Tool

The AQAT:RoB tool [43] is summarized in Multimedia Appendix 6. An easy-to-use version, which is both fillable and printable, is available on the AQAT website [43]. Textbox 1 presents the scope and boundaries of studies for which AQAT:RoB is applicable.

Textbox 1. **Utilization of Alberta Quality Assessment Tool: Risk of Bias (AQAT:RoB).**

Intended users: Anyone who wants to appraise the quality or risk of bias of studies in which there are human evaluations of large language model question-answer systems. It is especially important for researchers during the development and peer review of such studies and during the quality assessment stage of systematic literature reviews and meta-analyses. Other potential users include, but are not limited to, patients, health care providers, journal editors and reviewers, medical technology manufacturers, health system administrators, and policymakers.

Target studies: Any study that involves the human evaluation of large language models that provide answers to free-text questions including but not limited to:

- Patient-facing applications
  - Medical chatbots
  - Summary tools (eg, answer questions about imaging reports or doctors’ reports)
- Physician-facing applications
  - General medical chatbots
  - Large language model–based clinical decision support systems for physicians
  - Summary tools (eg, answer questions about patient charts)
- Research-based applications:
  - Case finding (eg, find participants based on electronic medical record or electronic health record data and provide justification)
  - Literature review (eg, analyze and summarize scientific literature)

The AQAT:RoB consists of 5 high-level domains (Questions, Reference Answers, LLM Answers, Evaluators, and Outcomes). These domains are subdivided into 9 subdomains. Each subdomain includes at least one “Support for Judgment” and at least one “Type of Bias.” Further descriptions of potential sources of bias and best methodological practices are outlined in the text below. In cases of missing, partial, or suboptimal reporting of a specific domain or subdomain, the rating of “unclear” should be assigned. Additional considerations regarding reporting and conflicts of interest are outlined in the *Additional Considerations* section.

#### Domain 1: Questions

##### Question Source

Supports for Judgment:

- If questions were created or generated specifically for the study, describe the method used to create the question dataset, including who created the questions and if the questions are reflective of the intended study objective.
- If questions were selected from an existing question source, adequately describe the source to allow an assessment of whether it addresses the intended research question.

The evaluation of LLM-QA models should be conducted against questions that reflect the intended use case, as deviations can introduce biases. When the deviation between intended use and the question source is substantial, the performance on the proxy task may not be generalizable to the stated application. To minimize this risk, the most effective approach is to source questions directly from the real-world use setting. If this is not possible, external data sources are often used to generate questions. In such cases, researchers must justify the degree to which these sources align with the study’s core research question and the tool’s intended application. For example, if a study evaluates a tool to be used by patients, but the questions are written by a research team of nonpatients, this could introduce bias, as the language and complexity of the questions may not be representative of the intended user. Furthermore, if questions were pulled from a preexisting source, it should be clearly stated if the test questions were included in the training data of the tested models, as performance may reflect memorization versus true model reasoning.

##### Question Selection

Support for Judgment:

- If questions were selected from an existing question source, describe the method used to select the questions from the original source (eg, random, consecutive, all, or by certain factors).

When selecting questions from an existing dataset, sampling can introduce bias as the selected questions may not be representative of the broader population of potential questions. For instance, a selection mechanism that favors questions of a specific length—such as those with a short, predefined character count, perhaps to minimize computational costs—would systematically exclude longer, more complex questions. Similarly, selecting from a small, nonrandom subset of available options could skew the results, as the chosen questions may not accurately reflect the diversity and range of questions encountered in the tool’s intended use case. Therefore, the method for question selection, such as random, purposive, or consecutive sampling, must be clearly reported and justified.

##### Question Manipulation

Supports for Judgment

- If any questions were manipulated from the original source, describe and justify the rationale for the manipulation.
- If any prompting was provided in addition to the index question, report the exact wording of the prompt(s).

Whether questions are created or extracted from existing sources, researchers may choose to manipulate them for various reasons. For example, slight variations might be introduced to test the model’s robustness and bias, assessing how stable its responses are to minor changes in phrasing. Such manipulations are generally less likely to introduce significant bias, as their purpose is to probe the model’s inherent stability rather than to alter the nature of the query. Conversely, questions might be manipulated to simplify them for processing by the model. A common example of this is the use of a system prompt that automatically extracts and restructures clinically relevant information before the model attempts to answer. This form of question manipulation, while potentially beneficial for processing, introduces a risk of bias because it may fundamentally alter the user’s original query. Or if researchers correct spelling, terminology, or grammatical errors, or split multipart patient questions into separate questions, these changes may augment the performance of the LLM-QA model but not necessarily reflect real-world performance. It is essential that researchers provide both transparency and justification for any question manipulation, as the process could alter the original intent of the question, thereby compromising the validity of the evaluation. Along with direct question manipulation, all prompts, including system prompts, which in and of themselves do not necessarily introduce bias, should be clearly described and should be standardized and stable throughout testing, as differences in prompts may lead to false or misleading performance outcomes.

### Domain 2: Reference Answers

#### Reference Answer Source

Supports for Judgment:

- If reference answers were generated specifically for the study, describe the method used to create the reference answer dataset, including who created the reference answers, and if the answers are reflective of a true reference standard.
- If reference answers were selected from an existing reference answer source, adequately describe the source to allow an assessment of whether it is reflective of a true reference standard.

Often, LLM-QA studies benchmark LLM outputs against reference answers. Bias may be introduced if the reference answers do not accurately reflect a “true” or expected standard. For instance, if reference answers were created by individuals with a different level of expertise or with a different format or standard than the true reference standard, the reference standard may not be valid. For example, if the research team created the reference answers to a higher or lower standard than real-world physician-level responses, the reference standard would be misaligned with the intended quality benchmark. A mismatch in language, structure, style, or level of detail between the study reference standard used and the “true” real-world reference standard can lead to biased results. As a single ground truth does not always exist in medicine, the selected reference standard should be decided a priori (eg, guideline-based or expert consensus-based) and described and justified.

#### Reference Answer Selection

Support for Judgment:

- If not all reference answers to a given question were used, describe the method by which reference answers were selected.

Just as with question selection, the process of selecting reference answers from a larger pool can introduce sampling bias. This bias occurs if the selection method systematically favors answers with certain qualities, making the final set of reference answers unrepresentative of the full range of possible correct responses. For example, if a question has multiple valid reference answers but researchers consistently choose those with a specific tone or level of detail, the evaluation will be skewed toward models that produce similar outputs. Therefore, it is critical to describe and justify the method used for selecting reference answers.

### Domain 3: LLM Answers

Support for Judgment:

- Describe how many answers were generated for each question and if not all answers were assessed, describe how answers were selected for assessment.

When evaluating language models, it is often prudent to generate multiple answers for a single question to assess the model’s stability or to explore the diversity of its outputs. In such instances, only evaluating a subset of the generated answers (eg, choosing the best one) may not be representative of the model’s typical performance, thereby leading to an inaccurate or misleading evaluation. Therefore, it is crucial for researchers to transparently describe and justify how many answers were generated for each question and, if not all of them were assessed, to detail the specific methodology used to select the answers for evaluation.

### Domain 4: Evaluators

#### Evaluator Selection

Support for Judgment

- Describe the method used to select evaluators, and assign evaluators to specific LLM qualities.

The selection of evaluators should reflect the intended real-world use and the required expertise to judge the domains being assessed. For example, having a physician evaluate the empathy of an LLM-QA’s outputs may not reflect how a patient would assess this domain. Likewise, it would not be appropriate for a patient to evaluate the accuracy of health information generated by an LLM-QA, as they would lack the appropriate expertise. As many studies assess multiple outcomes, more than one type of evaluator may be required for a given study (eg, physicians evaluate accuracy and patients rate readability). Furthermore, the demographic or professional characteristics of the evaluators should align with the intended user population of the LLM or chatbot. For example, if an LLM is designed for a general audience but its readability is evaluated exclusively by individuals with advanced academic degrees, the results may not accurately reflect how an average user would perceive the content.

#### Blinding of Evaluators

Support for Judgment:

- Describe all measures used, if any, to blind trial evaluators and researchers from knowledge of the answer source. Provide information relating to whether the intended blinding was effective.

The integrity of an evaluation can be compromised if evaluators are not blinded to the source of the answers they are assessing (reference standard vs LLM-generated) because evaluators’ preexisting beliefs, attitudes, or knowledge about a specific technology, such as AI, or even to a specific LLM model, can unconsciously influence their ratings. Thus, evaluators should be blinded to the answer source, and researchers should describe the blinding measures employed. Since naive blinding is not guaranteed to be effective given stylistic markers in LLM-generated text, authors should describe the steps taken to assess or verify the effectiveness of the blinding (eg, ask the evaluators if they could identify the AI-generated answer).

### Domain 5: Outcomes—Performance Metrics

Supports for Judgment:

- Describe specific metrics used for each outcome quality.
- Describes if desired outcomes were prespecified prior to conducting the study.

To minimize the risk of bias, 2 crucial steps should be taken. First, the desired outcomes or hypotheses of the study should be prespecified prior to conducting any analysis. Second, the metrics selected to measure each outcome must directly align with the stated goals of the study. For example, if the goal is to evaluate a chatbot’s ability to provide concise summaries of medical information, metrics should focus on conciseness and accuracy, rather than on secondary qualities, such as conversational tone or creativity. A misalignment between metrics and study goals introduces bias, as the evaluation would not accurately reflect the model’s performance on its intended task.

### Additional Considerations

Complete and transparent reporting is required to judge the risk of bias. Researchers must account for any instances of missing data and describe how missing data were handled. For example, if certain questions were too long for the model to process, or if the model failed to produce a response, these omissions should be explicitly noted and their potential impact on the evaluation should be discussed. Similarly, if human evaluators did not complete all of their annotations, it is important that this missingness is reported and ideally investigated, as these instances of missingness may not be random and could introduce bias if not accounted for. Study outcomes should be decided *a priori* and deviations should be described and justified. By reporting a subset of all measured outcomes or manipulating the analysis post hoc to achieve a different result (eg, by recategorizing certain groups, shifting the scale), a study may present a distorted or optimistic view of model performance. The use of appropriate reporting checklists is recommended.

Conflicts of interest (especially commercial interests) may introduce explicit or subconscious biases in the formulation of the problem, the execution of the analysis, or the interpretation of the results. If there are conflicts of interest (eg, authors funded or affiliated with model vendors), they must be disclosed and mitigated, if possible.

## Discussion

### Principal Findings

The AQAT:RoB tool was developed to standardize the quality assessment and specifically, the risk of bias assessments, of studies in which there are human evaluations of LLM-QA systems. This easy-to-use risk of bias assessment tool covers 5 major domains (Questions, Reference answers, LLM answers, Evaluators, Outcomes, Reporting, and Other), with a total of 9 potential sources of bias, each with 1‐2 support for judgment prompts and a list of types of potential bias. In our pilot validation by 4 assessors of 16 studies, the AQAT:RoB showed a substantial degree of agreement between internal validators blinded to the development process.

### Comparison to Prior Work

As demonstrated in Table 1, the AQAT:RoB assesses many important aspects of LLM-QA studies that are either not covered at all or only tangentially covered by other foundational risk of bias tools [4-7]. Most notably, Domain 1: Questions, a very important potential source of bias, is not addressed by any of the foundational tools. We also note that the panel explicitly discussed and voted to include conflict of interest in the AQAT:RoB, which is not always included in foundational tools, given the increasing commercialization of natural language processing research. The vast majority of computer science faculty at top schools have financial conflicts with industry [44], and the field of natural language processing is so reliant on industry artifacts to the point of being described as “captured” [45]. This is particularly relevant in evaluation works, as past peer-reviewed evaluations have then been used by the relevant industry party to claim that their models have been “independently audited” [46]. The foundational tools listed in Table 1 [4-7] either do not provide a threshold for determining overall risk of bias or use a “worst-of” approach, where the overall risk of bias is considered “High risk” if at least 1 domain is judged as “High risk.” We have chosen to leave the determination of overall risk of bias to the discretion of the user, as the threshold may be different depending on the intended use of the tool, although in most cases, a single domain being judged as “High risk” would likely result in an overall judgment of “High risk.”

**Table 1.** Comparison of Alberta Quality Assessment Tool: Risk of Bias (AQAT:RoB) and foundational risk of bias tools^a^.

;AQAT:RoB domain	RoB 2^b^ [4]	NOS^c^ [6]	QUADAS-2^d^ [5]	ROBINS-E^e^ [7]
Questions	x^f^	x	x	x
Reference answers	x	Domain 1: Selection Domain 2: Comparability	Domain 3: Reference standard Domain 4: Flow and timing	Domain 2: Risk of bias arising from measurement of the exposure
LLM^g^ answers	x	Domain 1: Selection Domain 2: Comparability	Domain 2: Index test or tests Domain 4: Flow and timing	Domain 2: Risk of bias arising from measurement of the exposure
Evaluators	Domain 4: Risk of bias in measurement of the outcome	Domain 1: Selection Domain 3: Outcomes	Domain 1: Patient selection	Domain 3: Risk of bias in selection of participants into the study (or into the analysis)
Outcomes	Domain 4: Risk of bias in measurement of the outcome	Domain 3: Outcomes	x	Domain 6: Risk of bias arising from measurement of the outcome

a This table compares the coverage of key domains determined to be important for assessing risk of bias in large language model question-answer studies by the Alberta Quality Assessment Tool: Risk of Bias (AQAT:RoB) compared with existing foundational risk of bias tools. No AQAT:RoB domains were covered adequately by other Foundational Risk of Bias tools. Terms in italics signify the closest domain found in the foundational tool.

b RoB 2: Cochrane Risk of Bias 2 tool.

c NOS: Newcastle-Ottawa Scale.

d QUADAS-2: Quality Assessment of Diagnostic Accuracy Studies 2.

e ROBINS-E: Risk of Bias in Non-randomized Studies - of Exposures.

f x: not covered by the tool.

g LLM: large language model.

The AQAT:RoB is the first risk of bias assessment tool specifically designed for studies of human-evaluated LLM-QA systems. Despite this, systematic reviews of LLM-QA studies have already been published [10,11], highlighting the urgency of the need for the AQAT:RoB. Previous AI evaluation frameworks primarily function as reporting checklists [9,12,13,15]. While these are invaluable for assessing transparency and reproducibility, they do not directly evaluate a study’s susceptibility to bias. For instance, a study may meticulously report every detail, yet still contain high-risk elements, such as a lack of blinding for answer sources, that could compromise its findings. Existing AI-specific quality assessment tools, such as PROBAST-AI [8] and APPRAISE-AI [9], are tailored for predictive machine learning models, which differ significantly from the evaluation needs of LLM-QA studies. Templin et al [47] introduced a useful 5-step framework for auditing LLMs, but not for assessing the validity of LLM-QA studies. Therefore, the AQAT:RoB addresses a critical void in the standardized evaluation of LLM-QA research.

Aiming to address the urgent need for a risk of bias assessment tool for this growing field and aided by our own existing [16] and recently published systematic reviews [10,11], we sought to develop the AQAT:RoB through a systematic, but pragmatic approach. With a highly engaged group of interdisciplinary experts, our pilot validation was able to achieve high interrater agreement after the first round of modified Delphi and consensus. In our pilot validation of 16 studies, the interrater reliability was on par with or better than that of existing foundational risk of bias tools. For example, studies have found that the RoB 2 demonstrated kappas consistent with “fair” agreement (0.21‐0.40) [48,49]. Another study found that while the interrater reliability across 6 risk of bias tools for nonrandomized studies varied widely, most demonstrated intraclass correlation coefficients in the substantial range, similar to the AQAT:RoB [50]. No studies have demonstrated the interrater reliability of existing RoB tools specifically to LLM-QA studies.

### Limitations

We recognize that there are limitations to the AQAT:RoB. First, while it was developed by a wide interdisciplinary group of experts and patient partners, most experts were from Alberta due to the nature of the AQAT collaborative, which includes the development of other quality assessment tools for AI-related studies, including the development of publicly available datasets, validated evaluation scales, and measurements and programs. In future updates of the AQAT:RoB, we plan to engage a more international group of partners. It will also be crucial for the AQAT:RoB to be extensively and externally validated by the broader international community of researchers [51]. Furthermore, the AQAT:RoB was developed with a medical focus and would require validation and adaptation for nonmedical studies of LLM-QA. Second, we recognize that this tool will need to evolve with the rapid development of LLM tools and related studies. Third, this tool was developed in English and evaluated only on English-language studies. In order to use this tool on non-English–language studies, it would ideally be translated and validated in other languages. Multilingual and non-English evaluations may require an expansion of the support for judgments to be considered when classifying the risk of bias. For example, if questions or reference answers are translated from English, they may not accurately reflect the distribution against which they will be evaluated (ie, native, nontranslated questions).

Finally, the pilot validation has limitations. It included only 16 studies, a relatively small sample, which may limit reliability estimates. While construct validity was supported by expert consensus and signaling questions that map directly to known mechanisms of bias, our pilot validation focused on interrater reliability, the most commonly reported evaluation metric for such tools. To further support construct validity, future evaluations that test concurrent and criterion validity are needed, although the lack of truly comparative tools and meta-analyses in this field limits the feasibility of these types of evaluations. Finally, reliance on an aggregate score for our a priori threshold makes it possible for domains with high agreement to compensate for domains with poor agreement. We acknowledge that agreement for the LLM Answer Selection domain was lower or less reliable than for the other domains and remains experimental, requiring future refinements after more extensive validation.

### Future Directions

The AQAT:RoB is a crucial next step toward standardizing the evaluation of LLM-QA studies in medicine. While we acknowledge that the tool will benefit from future refinements, more extensive validation (particularly external validation), and periodic updates to keep pace with evolving technology, we believe it currently fills an urgent need and critical gap. The immediate application of this tool will enable researchers, clinicians, and policymakers to more effectively and rigorously assess the validity of LLM-based studies, thereby ensuring that real-world applications of this technology are built on a solid foundation of reliable evidence.

### Conclusions

The AQAT:RoB demonstrates promising initial reliability for assessing the validity or risk of bias of LLM-QA studies.

## Supplementary material

10.2196/87057 Multimedia Appendix 1 Search strategy for the large language model (LLM) patient education systematic literature review.

10.2196/87057 Multimedia Appendix 2 PRISMA flow diagram of unpublished systematic literature review of studies evaluating large language models (LLMs) for patient-facing health information published between July 2013 and March 2024.

10.2196/87057 Multimedia Appendix 3 Large language model (LLM) patient education systematic literature review list of studies reviewed.

10.2196/87057 Multimedia Appendix 4 Studies used in the pilot validation set (n=16 studies).

10.2196/87057 Multimedia Appendix 5 Pilot validation dataset. Sixteen papers were each assessed by 2 evaluators for a total of 32 evaluations.

10.2196/87057 Multimedia Appendix 6 Alberta Quality Assessment Tool: Risk of Bias (AQAT:RoB).

## Abbreviations

AI: artificial intelligence
AQAT:RoB: Alberta Quality Assessment Tool: Risk of Bias
CHART: Chatbot Assessment Reporting Tool
LLM: large language model
LLM-QA: LLM question-answer system
NOS: Newcastle-Ottawa Scale
PROBAST-AI: Prediction model Risk of Bias Assessment Tool
QUADAS-2: Quality Assessment of Diagnostic Accuracy Studies 2
RoB 2: Cochrane Risk of Bias 2 tool
ROBINS-E: Risk of Bias in Non-randomized Studies - of Exposures tool
