# Supplementary material for: The Alberta Quality Assessment Tool: Risk of Bias (AQAT:RoB) for the Evaluation of Medical Large Language Model Question-Answer Studies: Development and Pilot Validation
Source: J Med Internet Res. 2026 Apr 8;28:e87057. doi: 10.2196/87057 (PMC13061365; doi:10.2196/87057)
Supplement: Multimedia Appendix 1 [file jmir-v28-e87057-s001.docx]

**Appendix 1. Search Strategy LLM Patient Education Systematic Literature Review**

Database(s): **Ovid MEDLINE(R) ALL**
Search Strategy:

| **#** | **Searches** |
| --- | --- |
| 1 | ((artificial intelligence or AI) adj2 generat*).mp. |
| 2 | ((large or natural or generative or machine or deep learning) adj3 (language or text) adj3 model*).mp. |
| 3 | (chatgpt or chatbot* or BERT or XLNet or GPT-2 or GPT-3* or GPTNeo or GPT-J or Megatron-Turing NLG or Ernie or Claude or GLaM or Gopher or LaMDA or GPT-NEoX or Chinchilla or PaLM or OPT or YaLM 100B or Minerva or BLOOM or Galactica or AlexaTM or LLaMA or GPT-4 or Cerebras-GPT or Falcon or BloombergGPT or PanGu-E or OpenAssistant or PaLM or YouChat or NeevaAI or Perplexity or Chatsonic or Bing Chat or Bard or EinsteinGPT or SlackGPT or BioGPT or GPT-JT).mp. |
| 4 | Generative pre trained transformer*.mp. |
| 5 | exp Patient Education as Topic/ |
| 6 | consumer health information/ or health literacy/ |
| 7 | exp Health Education/ |
| 8 | exp Health Promotion/ |
| 9 | Patient Participation/ |
| 10 | Consumer Health Informatics/ |
| 11 | health communication/ or information dissemination/ or information literacy/ |
| 12 | exp Access to Information/ |
| 13 | ((patient* or consumer* or lay* or health or medical) adj3 (educat* or information or literac* or engag* or question* or support* or counsel* or advice or advis* or instruct* or knowledge or communicat*)).mp. |
| 14 | 5 or 6 or 7 or 8 or 9 or 10 or 11 or 12 or 13 |
| 15 | 1 or 2 or 3 or 4 |
| 16 | 14 and 15 |
| 17 | limit 16 to yr="2013 -Current" |

Database(s): **Embase**
Search Strategy:

| **#** | **Searches** |
| --- | --- |
| 1 | ((artificial intelligence or AI) adj2 generat*).mp. |
| 2 | ((large or natural or generative or machine or deep learning) adj3 (language or text) adj3 model*).mp. |
| 3 | (chatgpt or chatbot* or BERT or XLNet or GPT-2 or GPT-3* or GPTNeo or GPT-J or Megatron-Turing NLG or Ernie or Claude or GLaM or Gopher or LaMDA or GPT-NEoX or Chinchilla or PaLM or OPT or YaLM 100B or Minerva or BLOOM or Galactica or AlexaTM or LLaMA or GPT-4 or Cerebras-GPT or Falcon or BloombergGPT or PanGu-E or OpenAssistant or PaLM or YouChat or NeevaAI or Perplexity or Chatsonic or Bing Chat or Bard or EinsteinGPT or SlackGPT or BioGPT or GPT-JT).mp. |
| 4 | Generative pre trained transformer*.mp. |
| 5 | exp health education/ or exp health literacy/ or exp health promotion/ or patient education/ |
| 6 | exp patient participation/ |
| 7 | consumer health informatics/ |
| 8 | exp information dissemination/ or exp information literacy/ |
| 9 | exp access to information/ |
| 10 | ((patient* or consumer* or lay* or health or medical) adj3 (educat* or information or literac* or engag* or question* or support* or counsel* or advice or advis* or instruct* or knowledge or communicat*)).mp. |
| 11 | 1 or 2 or 3 or 4 |
| 12 | 5 or 6 or 7 or 8 or 9 or 10 |
| 13 | 11 and 12 |
| 14 | limit 13 to yr="2013 -Current" |

**APA PsycInfo**
1 ((artificial intelligence or AI) adj2 generat*).mp.
2 ((large or natural or generative or machine or deep learning) adj3 (language or text) adj3 model*).mp.
3 (chatgpt or chatbot* or BERT or XLNet or GPT-2 or GPT-3* or GPTNeo or GPT-J or Megatron-Turing NLG or Ernie or Claude or GLaM or Gopher or LaMDA or GPT-NEoX or Chinchilla or PaLM or OPT or YaLM 100B or Minerva or BLOOM or Galactica or AlexaTM or LLaMA or GPT-4 or Cerebras-GPT or Falcon or BloombergGPT or PanGu-E or OpenAssistant or PaLM or YouChat or NeevaAI or Perplexity or Chatsonic or Bing Chat or Bard or EinsteinGPT or SlackGPT or BioGPT or GPT-JT or "Generative pre trained transformer*").mp.
4 1 or 2 or 3
5 client education/
6 health knowledge/
7 exp health information/
8 exp health literacy/
9 exp health education/
10 exp health promotion/
11 information literacy/
12 information dissemination/
13 information seeking/
14 ((patient* or consumer* or lay* or health or medical) adj3 (educat* or information or literac* or engag* or question* or support* or counsel* or advice or advis* or instruct* or knowledge or communicat*)).mp.
15 5 or 6 or 7 or 8 or 9 or 10 or 11 or 12 or 13 or 14
16 4 and 15
17 limit 16 to yr="2013 -Current"

**Web of Science Core Collection**

Limit search from 2013 to Current

TS=((("artificial intelligence" or AI) NEAR/2 generat*) OR ((large or natural or generative or machine or "deep learning") NEAR/3 (language or text) NEAR/3 model*) OR chatgpt or chatbot* or BERT or XLNet or GPT-2 or GPT-3* or GPTNeo or GPT-J or "Megatron-Turing NLG" or Ernie or Claude or GLaM or Gopher or LaMDA or GPT-NEoX or Chinchilla or PaLM or OPT or "YaLM 100B" or Minerva or BLOOM or Galactica or AlexaTM or LLaMA or GPT-4 or Cerebras-GPT or Falcon or BloombergGPT or PanGu-E or OpenAssistant or PaLM or YouChat or NeevaAI or Perplexity or Chatsonic or "Bing Chat" or Bard or EinsteinGPT or SlackGPT or BioGPT or GPT-JT or "Generative pre trained transformer*") AND

(KP=("health promotion" OR "information literacy" OR "Information Dissemination" OR "patient participation" OR "health communication") OR TS=((patient* or consumer* or lay* or health or medical) NEAR/3 (educat* or information or literac* or engag* or question* or support* or counsel* or advice or advis* or instruct* or knowledge or communicat*)))

**Google Scholars**

Limit search from 2013 to Current

Patient health information "Generative Artificial Intelligence" OR "generative AI" OR ChatGPT OR "Large language Model" OR Chatbot
