# Supplementary figures and images for: The Alberta Quality Assessment Tool: Risk of Bias (AQAT:RoB) for the Evaluation of Medical Large Language Model Question-Answer Studies: Development and Pilot Validation
Source: J Med Internet Res. 2026 Apr 8;28:e87057. doi: 10.2196/87057 (PMC13061365; doi:10.2196/87057)

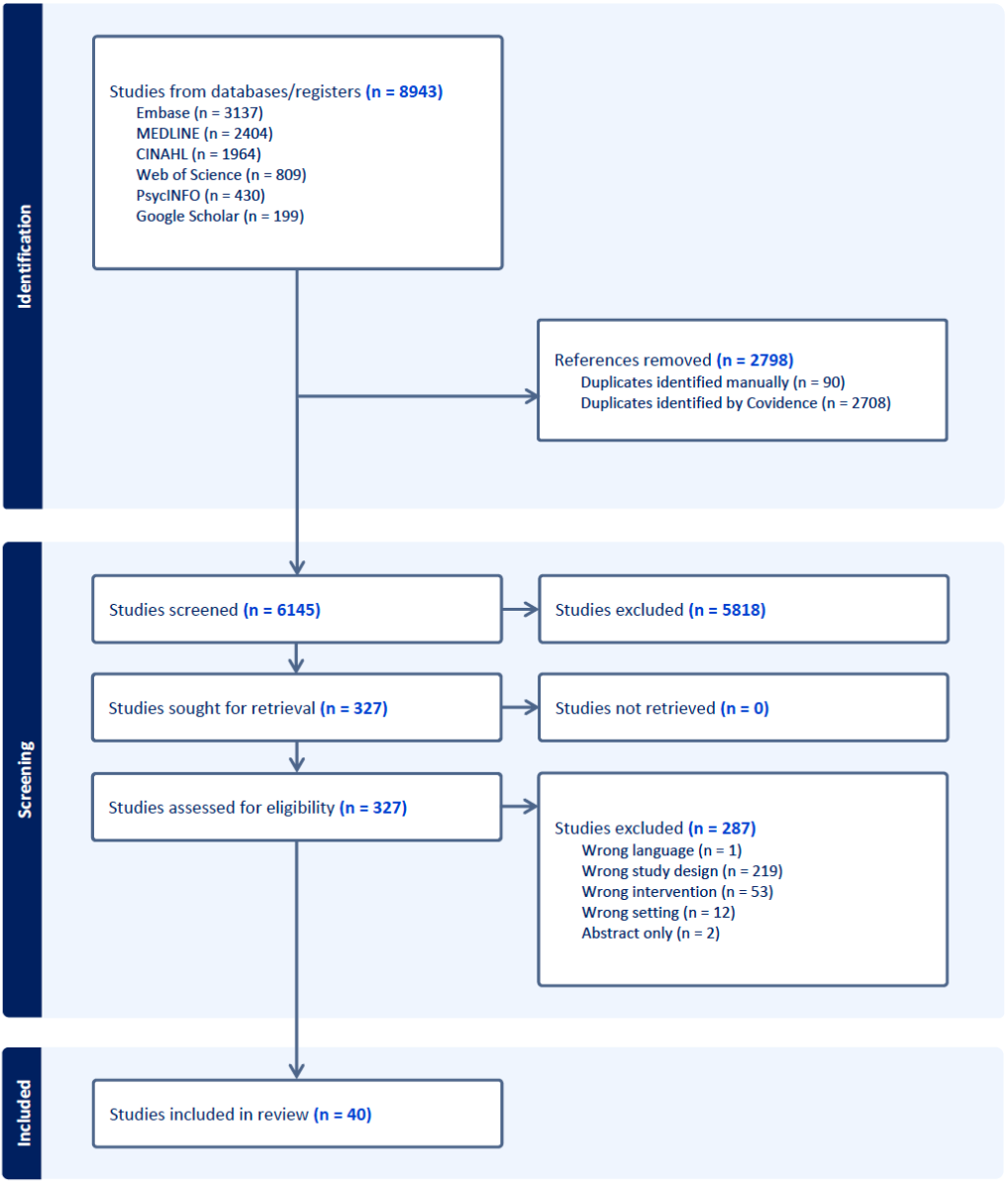

Supplement: Multimedia Appendix 2 [file jmir-v28-e87057-s002.png]
