# Supplementary material for: The Alberta Quality Assessment Tool: Risk of Bias (AQAT:RoB) for the Evaluation of Medical Large Language Model Question-Answer Studies: Development and Pilot Validation
Source: J Med Internet Res. 2026 Apr 8;28:e87057. doi: 10.2196/87057 (PMC13061365; doi:10.2196/87057)
Supplement: Multimedia Appendix 3 [file jmir-v28-e87057-s003.pdf]

Appendix 3. LLM Patient Education Systematic Literature Review List of Studies Reviewed

Study Author Publication Year	Title
Verran 2024	Artificial intelligence-generated patient information leaflets: a comparison of contents according to British Association of Dermatologists standards
Yucel 2023	Can artificial intelligence provide accurate and reliable answers to cancer patients' questions? Comparison of chatbots based on the ESMO Patient Guide about cancer pain
Collins 2023	Using AI-Generated Patient Information Sheets on Colonoscopies to Improve Doctor-Patient Communication
Buzzaccarini 2023	Exploring the Potential of Artificial Intelligence in Providing Infertility Consultations and Answers: can it replace the infertility specialist? A hybrid study
Taylor 2023	Can Sexual Health Clinicians Be Replaced By Robots? Utility Of Artificial Intelligence Platforms To Give Sexual Health Advice
Yazici 2023	2066P Evaluation of the quality and reliability of ChatGPT and Perplexity's responses about rectal cancer
Liu 2023	Leveraging Large Language Models for Generating Responses to Patient Messages.
Haidar 2023	AI-Generated Information for Vascular Patients: Assessing the Standard of Procedure-Specific Information Provided by the ChatGPT AI-Language Model.
Thia 2024	ChatGPT: Is This Patient Education Tool for Urological Malignancies Readable for the General Population?.
Vallurupalli 2024	Validation of ChatGPT 3.5 as a Tool to Optimize Readability of Patient-facing Craniofacial Education Materials.
Liu 2023	Consulting the Digital Doctor: Google Versus ChatGPT as Sources of Information on Breast Implant-Associated Anaplastic Large Cell Lymphoma and Breast Implant Illness.
Moosvi 2023	Readability, accuracy, and appropriateness of ChatGPT 4.0 responses for use in patient education materials for Condyloma acuminatum.
Solli 2023	Analysis of ChatGPT responses to patient-oriented questions on common ophthalmic procedures.
Verran 2024	AI-generated Patient Information Leaflets: a comparison of PIL contents to BAD standards.
Chaker 2024	Easing the Burden on Caregivers- Applications of Artificial Intelligence for Physicians and Caregivers of Children with Cleft Lip and Palate.
Ye 2023	Doctor Versus Artificial Intelligence: Patient and Physician Evaluation of Large Language Model Responses to Rheumatology Patient Questions in a Cross-Sectional Study.
Cappellani 2024	Reliability and accuracy of artificial intelligence ChatGPT in providing information on ophthalmic diseases and management to patients.
Hristidis 2023	ChatGPT vs Google for Queries Related to Dementia and Other Cognitive Decline: Comparison of Results.
Sezgin 2023	Clinical Accuracy of Large Language Models and Google Search Responses to Postpartum Depression Questions: Cross-Sectional Study.
Hung 2023	Comparison of Patient Education Materials Generated by Chat Generative

	Pre-Trained Transformer Versus Experts: An Innovative Way to Increase Readability of Patient Education Materials.
Liu 2023	Dr. ChatGPT will see you now: How do Google and ChatGPT compare in answering patient questions on breast reconstruction?.
Gabriel 2023	The utility of the ChatGPT artificial intelligence tool for patient education and enquiry in robotic radical prostatectomy.
Crook 2023	Evaluation of Online Artificial Intelligence-Generated Information on Common Hand Procedures.
Wei 2024	Answering head and neck cancer questions: An assessment of ChatGPT responses.
Nanji 2024	Evaluation of postoperative ophthalmology patient instructions from ChatGPT and Google Search.
Kianian 2024	Can ChatGPT Aid Clinicians in Educating Patients on the Surgical Management of Glaucoma?.
Peng 2024	Evaluating AI in medicine: a comparative analysis of expert and ChatGPT responses to colorectal cancer questions.
Bernstein 2023	Comparison of Ophthalmologist and Large Language Model Chatbot Responses to Online Patient Eye Care Questions.
Kim 2024	Assessing the performance of ChatGPT's responses to questions related to epilepsy: A cross-sectional study on natural language processing and medical information retrieval.
Bitar 2022	Increasing Women's Knowledge about HPV Using BERT Text Summarization: An Online Randomized Study
Padovan 2023	ChatGPT in Occupational Medicine: A Comparative Study with Human Experts
Nov 2023	Putting ChatGPT's Medical Advice to the (Turing) Test
McCarthy 2023	Evaluation of an Artificial Intelligence Chatbot for Delivery of Interventional Radiology Patient Education Material: A Comparison with Societal Website Content
Krittanawong 2023	Assessing the Potential of ChatGPT for Patient Education in Cardiac Catheterization Care
Coskun 2023	CAN CHATGPT, AN ARTIFICIAL INTELLIGENCE LANGUAGE MODEL, PROVIDE ACCURATE AND HIGH-QUALITY PATIENT INFORMATION ON PROSTATE CANCER?
Chiesa-Estomba 2023	Exploring the potential of Chat-GPT as a supportive tool for sialendoscopy clinical decision making and patient information support
Chervenak 2023	The promise and peril of using a large language model to obtain clinical information: ChatGPT performs strongly as a fertility counseling tool with limitations
Chen 2023	The utility of ChatGPT for cancer treatment information
Ayoub 2023	Comparison between ChatGPT and Google Search as Sources of Postoperative Patient Instructions
Ayers 2023	Comparing Physician and Artificial Intelligence Chatbot Responses to Patient Questions Posted to a Public Social Media Forum
