# Supplementary material for: The Alberta Quality Assessment Tool: Risk of Bias (AQAT:RoB) for the Evaluation of Medical Large Language Model Question-Answer Studies: Development and Pilot Validation
Source: J Med Internet Res. 2026 Apr 8;28:e87057. doi: 10.2196/87057 (PMC13061365; doi:10.2196/87057)
Supplement: Multimedia Appendix 4 [file jmir-v28-e87057-s004.pdf]

Appendix 4. Studies used in the pilot validation set (n=16 studies)

1. Bernstein IA, Zhang YV, Govil D, Majid I, Chang RT, Sun Y, Shue A, Chou JC, Schehlein E, Christopher KL, Groth SL, Ludwig C, Wang SY. Comparison of ophthalmologist and large language model chatbot responses to online patient eye care questions. JAMA Netw Open; 2023 Aug 1;6(8):e2330320. PMID:37606922
2. Cappellani F, Card KR, Shields CL, Pulido JS, Haller JA. Reliability and accuracy of artificial intelligence ChatGPT in providing information on ophthalmic diseases and management to patients. EYE; 2024 May;38(7):1368–1373. PMID:38245622
3. Chaker SC, Hung Y-C, Saad M, Golinko MS, Galdyn IA. Easing the burden on caregivers- applications of artificial intelligence for physicians and caregivers of children with cleft lip and palate. Cleft Palate Craniofac J; 2025 Apr;62(4):574–587. PMID:38178785
4. Chen S, Kann B, Foote M, Aerts H, Savova G, Mak R, Bitterman D. The utility of ChatGPT for cancer treatment information. medRxiv 2023 Mar 23; doi: 10.1101/2023.03.16.23287316
5. Chervenak J, Lieman H, Blanco-Breindel M, Jindal S. The promise and peril of using a large language model to obtain clinical information: ChatGPT performs strongly as a fertility counseling tool with limitations. Fertil Steril; 2023 Sep;120(3 Pt 2):575–583. PMID:37217092
6. Coskun B, Ocakoglu G, Yetemen M, Kaygisiz O. Can ChatGPT, an artificial intelligence language model, provide accurate and high-quality patient information on prostate cancer? Urology; 2023 Oct;180:35–58. PMID:37406864
7. Gabriel J, Shafik L, Alanbuki A, Larner T. The utility of the ChatGPT artificial intelligence tool for patient education and enquiry in robotic radical prostatectomy. Int Urol Nephrol; 2023 Nov;55(11):2717–2732. PMID:37528247
8. Liu HY, Alessandri Bonetti M, Jeong T, Pandya S, Nguyen VT, Egro FM. Dr. ChatGPT will see you now: How do Google and ChatGPT compare in answering patient questions on breast reconstruction? J Plast Reconstr Aesthet Surg; 2023 Oct;85:488–497. PMID:37598590

9. Kianian R, Sun D, Giaconi J. Can ChatGPT aid clinicians in educating patients on the surgical management of glaucoma? J Glaucoma; 2024 Feb 1;33(2):94–100. PMID:38031276
10. McCarthy CJ, Berkowitz S, Ramalingam V, Ahmed M. Evaluation of an artificial intelligence chatbot for delivery of IR patient education material: A comparison with societal website content. J Vasc Interv Radiol; 2023 Oct;34(10):1760–1768.e32. PMID:37330210
11. Padovan M, Cosci B, Petillo A, Nerli G, Porciatti F, Scarinci S, Carlucci F, Dell'Amico L, Meliani N, Necciari G, Lucisano VC, Marino R, Foddis R, Palla A. ChatGPT in occupational medicine: A comparative study with human experts. Bioengineering; 2024 Jan 6;11(1). PMID:38247934
12. Thia I, Saluja M. ChatGPT: Is this patient education tool for urological malignancies readable for the general population? Res Rep Urol 2024 Jan 16;16:31–37. PMID:38259300
13. Mayo-Yáñez M, Lechien JR, Maria-Saibene A, Vaira LA, Maniaci A, Chiesa-Estomba CM. Examining the performance of ChatGPT 3.5 and Microsoft Copilot in otolaryngology: A comparative study with otolaryngologists' evaluation. Indian J Otolaryngol Head Neck Surg; 2024 Aug;76(4):3465–3469. PMID:39130248
14. Pressman SM, Borna S, Gomez-Cabello CA, Haider SA, Forte AJ. AI in hand surgery: Assessing large language models in the classification and management of hand injuries. J Clin Med; 2024 May 11;13(10):2832. PMID:38792374
15. Mesnier J, Suc G, Sayah N, Abtan J, Steg PG. Relevance of medical information obtained from ChatGPT: Are large language models friends or foes? Arch Cardiovasc Dis; 2023 Oct;116(10):485–486. PMID:37718185
16. Rizwan A, Sadiq T. The use of AI in diagnosing diseases and providing management plans: A consultation on cardiovascular disorders with ChatGPT. Cureus; 2023 Aug 7;15(8):e43106. PMID:37692649
