# Supplementary material for: The Alberta Quality Assessment Tool: Risk of Bias (AQAT:RoB) for the Evaluation of Medical Large Language Model Question-Answer Studies: Development and Pilot Validation
Source: J Med Internet Res. 2026 Apr 8;28:e87057. doi: 10.2196/87057 (PMC13061365; doi:10.2196/87057)
Supplement: Multimedia Appendix 6 [file jmir-v28-e87057-s006.docx]

| **Potential source of bias** | **Support for judgement** | **Type of bias** | **Assessment of risk of bias** (low, high, or unclear) |
| --- | --- | --- | --- |
| **Questions** | | | |
| Question source | If questions were created/generated specifically for the study, describe the method used to create the question dataset, including who created the questions and if the questions are reflective of the intended study objective  If questions were selected from an existing question source, adequately describe the source to allow an assessment of whether it addresses the intended research question | Expectancy bias due to creation of questions reflective of researcher's expectations  Representation bias due to question source not being representative of the target population  Construct-validity bias due to questions not matching the research aim |  |
| Question selection | If questions were selected from an existing question source, describe the method used to select the questions from the original source (eg, random, consecutive, all, or by certain factors) | Sampling bias due to question not being representative of the intended setting |  |
| Question manipulation | If any questions were manipulated from the original source, describe and justify the rationale for the manipulation.  Or if any prompting was provided in addition to the index question, report the exact wording of the prompt(s) | Construct-validity bias due to questions not matching the research aim  Expectancy bias due to question manipulation that may reflect researcher’s expectations |  |
| **Reference answers** | | | |
| Reference answer source | If reference answers were generated specifically for the study, describe the method used to create the reference answer dataset, including who created the reference answers and if the answers are reflective of a true reference standard  If reference answers were selected from an existing reference answer source, adequately describe the source to allow an assessment of whether it is reflective of a true reference standard | Construct-validity bias due to answers not matching the true reference standard study was designed to evaluate  Representation bias due to question source not being representative of the target population |  |
| Reference answer selection | If not all reference answers to a given question were used, describe the method by which reference answers were selected | Sampling bias due to answers not being representative of the intended setting |  |
| **LLM answers** | | |  |
| LLM answer selection | Describe how many answers were generated for each question and if not all answers were assessed, describe how answers were selected for assessment | Sampling bias due to answers not being representative of all LLM answers |  |
| **Evaluators** | | | |
| Evaluator selection | Describe the method used to select evaluators, and assign evaluators to specific LLM qualities | Sampling bias due to evaluators not being representative of the intended setting  Observation bias due to inadequate or inappropriate evaluator expertise for a specific LLM quality |  |
| Blinding of evaluators | Describe all measures used, if any, to blind trial evaluators and researchers from knowledge of the answer source. Provide information relating to whether the intended blinding was effective. | Detection bias due to knowledge of the answer source |  |
| **Outcomes** | | | |
| Performance metrics | Describe specific metrics used for each outcome quality  Describes if desired outcomes were pre-specified prior to conducting the study. | Measurement bias due to the LLM qualities or evaluation metrics not matching the research aim |  |

*Note:* AQAT:RoB was developed through a modified-Delphi process and consensus meeting of medical AI experts in Alberta, Canada in May 2025, with a second modified-Delphi in February 2026. The tool achieved a percent agreement of 86.1% and a Cohen’s Kappa of 0.70. The domain LLM Answer Selection had an agreement of 68.8% and Kappa of 0.30 and remains experimental.
